# Genome-Wide Identification and Transcriptional Regulation of Aquaporin Genes in Bread Wheat (*Triticum aestivum* L.) under Water Stress

**DOI:** 10.3390/genes9100497

**Published:** 2018-10-15

**Authors:** José Madrid-Espinoza, Nidia Brunel-Saldias, Fernando P. Guerra, Adelina Gutiérrez, Alejandro del Pozo

**Affiliations:** 1 Laboratorio de Genómica Funcional, Instituto de Ciencias Biológicas, Universidad de Talca, Talca 3460000, Chile; 2 Centro de Mejoramiento Genético y Fenómica Vegetal, Facultad de Ciencias Agrarias, Universidad de Talca, Talca 3460000, Chile; nidiabrunel@gmail.com (N.B-S.); adelpozo@utalca.cl (A.d.P.); 3 PIEI Adaptación de la Agricultura al Cambio Climático (A2C2), Universidad de Talca, Talca 3460000, Chile; 4 Laboratorio de Genética y Biotecnología Forestal, Instituto de Ciencias Biológicas, Universidad de Talca, Talca 3460000, Chile; fguerra@utalca.cl (F.P.G.); adgutierrez@utalca.cl (A.G.)

**Keywords:** wheat, aquaporins, gene expression, evolution, water stress responses

## Abstract

Aquaporins (AQPs) are transmembrane proteins essential for controlling the flow of water and other molecules required for development and stress tolerance in plants, including important crop species such as wheat (*Triticum aestivum*). In this study, we utilized a genomic approach for analyzing the information about AQPs available in public databases to characterize their structure and function. Furthermore, we validated the expression of a suite of AQP genes, at the transcriptional level, including accessions with contrasting responses to drought, different organs and water stress levels. We found 65 new AQP genes, from which 60% are copies expanded by polyploidization. Sequence analysis of the AQP genes showed that the purifying selection pressure acted on duplicate genes, which was related to a high conservation of the functions. This situation contrasted with the expression patterns observed for different organs, developmental stages or genotypes under water deficit conditions, which indicated functional divergence at transcription. Expression analyses on contrasting genotypes showed high gene transcription from Tonoplast Intrinsic Protein 1 (TIP1) and 2 (TIP2), and Plasma Membrane Intrinsic Protein 1 (PIP1) and 2 (PIP2) subfamilies in roots and from TIP1 and PIP1 subfamilies in leaves. Interestingly, during severe drought stress, 4 TIP genes analyzed in leaves of the tolerant accession reached up to 15-fold the level observed at the susceptible genotype, suggesting a positive relationship with drought tolerance. The obtained results extend our understanding of the structure and function of AQPs, particularly under water stress conditions.

## 1. Introduction

*Triticum aestivum*—bread wheat or common wheat is the world’s largest crop (~220 million ha) and it is cultivated in a wide variety of environments, many of them with severe water limitations [1,2]. The development of drought-tolerant and water-use efficient wheat genotypes is of global interest because stress by water deficit and/or drought can drastically reduce yield and grain quality [2]. In Mediterranean climate regions, wheat and other annual crops are exposed to water deficit during grain filling, leading to what has been called ‘terminal drought stress’ [3].

Tolerance to water deficit is a complex trait under polygenic control. In wheat, water uptake and hydraulic conductivity by roots are strongly influenced by aquaporins (AQPs) [4,5]. These proteins are located in the cell membranes and form channels which mediate the absorption and movement of water between the tissues and organs of the plant [6]. Recent studies, aimed at optimizing the efficient use of water and nutrients, have highlighted the functional importance of this type of channel, codified by a multigenic family [7]. Aquaporins belong to the superfamily of major intrinsic proteins (MIPs) and they are essential to facilitate the bidirectional flow of water or other uncharged small molecules, such as urea, carbon dioxide, boric acid or silicon, through biological membranes [8,9,10,11]. Structurally, they have six trans-membrane (TM) alpha helices and two short TM alpha helices that have a conserved NPA domain (Asp-Pro-Ala) and a selectivity pore ar/R (aromatic/Arg) that regulates the passage of water [12]. According to their intracellular location and phylogenetic distribution, AQPs have been divided into seven families. Within these, five are well known: intrinsic plasma membrane (PIP), intrinsic tonoplast (TIP), intrinsic nodulin 26-like (NIP), intrinsic small (SIP) and intrinsic uncharacterized (XIP) [13].

The role of AQPs in increasing membrane permeability to facilitate the mobilization of water and other small molecules essential for plant growth and development has been well documented [6]. AQPs are important in many processes for example, seed germination [14], leaf hydraulic and stomatal conductance [15] and cell elongation [16]. Their physiological role has been demonstrated in several studies on different species. In *Gossypium hirsutum*, *GhPIP2* is relevant in the rapid formation of fibers [17]. In *Arabidopsis thaliana*, *AtNIP5;1* is essential for cell elongation and fertility processes, allowing the mobilization of boron [10]. The ectopic expression of an antisense construct targeting *Brassica napus BnPIP1* in tobacco causes morphological deformations [18]. The activity of AQP is regulated by the phosphorylation mediated by kinases or phosphatases [13,19].

Aquaporin genes are differentially expressed in response to internal signals or environmental stresses [20]. In *Solanum lycopersicum*, 23 of 32 AQP genes modulated their expression in different tissues and some of them did this in a specific way [21]. Different genotypes of *Musa acuminata* grown under water stress also showed differential expression patterns with respect to PIP genes such as *MaPIP1-7*, *MaPIP2-6* and *MaPIP2-10*, among others [22]. In *Hordeum vulgare*, the expression levels of PIP and TIP genes were higher than that of NIP and SIP [23], similarly to that observed in *A. thaliana* [24]. Salinity in *H. vulgare* differentially modulated AQPs, especially PIP genes, with differences between tissues and even between sensitive and tolerant genotypes [24]. Other studies have shown that ectopic expression of *TaAQP7*, *TaAQP8* and *TaNIP* genes in transgenic tobacco plants and *A. thaliana* increased tolerance to water and salt stress in both species [25,26,27]. Equally, ectopic expression of *VfPIP1* [28], *MaPIP1* [29], *PgTIP1* [30] and *OsPIP1-1* [31] increased drought tolerance in transgenic *A. thaliana* plants and a similar effect was observed with *SlPIP2;2* in *S. lycopersicum* [32].

More than 1200 AQP genes have been identified in 31 plant species, including 34 in *Oryza sativa*, 35 in *A. thaliana*, 54 in *Populus trichocarpa*, 31 in *Zea mays* and 40 in *H. vulgare* [33]. In silico methods and databases of sequenced genomes have facilitated the characterization of these genes in several species such as *S. lycopersicum*, *M. acuminata* and *A. thaliana* [21,22,34]. In wheat, a comparative analysis and synteny with *O. sativa* AQPs revealed only 24 PIP and 11 TIP genes [35]. A subsequent in silico analysis added 6 PIP, 1 TIP, 4 NIP and 2 SIP genes to the list [36]. Currently, new genomic information resources are available, including the first version of the wheat genome sequence (http://www.wheatgenome.org/), allowing the study of the genes encoding these proteins [37]. However, additional research is required for organizing the accumulated information generated by these different sources and using it to deepen the analysis of the structure and function of these proteins.

*Triticum aestivum* is a hexaploid species with genomes A, B and D. Genetic analyses have established that *Triticum urartu* (A-genome donor) and *Aegilops speltoides* (B-genome donor) are the progenitors of the allotetraploid wild emmer wheat [*Triticum turgidum* ssp. *dicoccoides* (körn.) Thell] (2n = 4x = 28; AABB), which subsequently hybridized with *Aegilops tauschii* (D-genome donor) to form the current genome composition of bread wheat (2n = 6x = 42; AABBDD) [38]. These events have modified the number and/or levels of expression of different gene families, such as *EXP* expansins, *SWEET* sugar transporters, *MAPK* and *MAPKK* kinases, *PHT1* phosphate transporters, among others, implicating a change in their biological role [39,40,41,42].

In this study, we carried out a complete genome analysis utilizing the information about AQPs available in public databases. We analyzed their amino acid sequences, physico-chemical properties, gene structure and synteny. Furthermore, we explored the transcript profiles of a suite of AQP genes using accessions with contrasting responses to drought, different organs and water stress levels, to test the relationship between differential expression of those genes and a differential response to water deficit. The evolutionary history of duplicated genes and the spatio-temporal expression patterns of the AQP family, suggests possible divergences at the functional level. We conclude that these functional differences could be involved in the drought tolerance mechanism of wheat plants.

## 2. Materials and Methods

### 2.1. Identification and Phylogenetic Analysis of Aquaporins in Wheat

Sequences of approximately 99.000 genes in the wheat genome were obtained from EnsemblPlants [43] to establish a local database and determine genes corresponding to AQPs. Aquaporins encoded in the genome were identified using the MIP aquaporin-specific domains (Pfam PF00230) [44] as a profile for hidden Markov model (HMM) chains with a cut-off value of 1× 10^−10^ in the HMMER v3.1 software [45]. In addition, an automatic search and sequence annotation analysis was carried out using the HAMAP-Scan software [46]. Manual validation of each sequence was done by comparative analysis with the BLASTp databases (https://blast.ncbi.nlm.nih.gov), Pfam and CDD [47], sequences lacking the conserved amino acids in the selectivity pore and those encoding truncated proteins were discarded, allowing the elimination of false positives. The complete length of each protein sequence of wheat AQPs and those of model species such as *A. thaliana*, *O. sativa* and *P. trichocarpa* [48], were used as templates for multiple alignment with ClustalO [49]. The phylogenetic tree was constructed using MEGA7 software [50] and the neighbor-joining method with 5000 iterations bootstrap.

### 2.2. Analysis of the DNA Sequence and Predicted Proteins

The exon-intron structure of the AQP genes was obtained from the GSDS software [48]. The conserved motifs in the amino acid sequence were identified using the web tool MEME [51] and analyzed with InterPro Scan 5 [52]. The isoelectric point (pI) and molecular weight (MW) were predicted with ExPaSy’s Compute pI/Mw tool [53], the transmembrane domains of each protein were predicted using the TMHMM Server v. 2.0 [54] and sub-cellular localization using the WoLF PSORT tool [55].

### 2.3. Expansion Pattern and Collinearity Analyses for the Aquaporin Family

The AQP genes were mapped in the genome of wheat by identifying their A, B and D sub-genomes position provided by the EnsemblPlants database. Groups of AQP genes were classified as paralogs in the wheat genome according to the same database. To establish the expansion pattern, those duplicate AQP genes that were in distinct sub-genomes of wheat according to the ENSEMBL Gene Tree tool were regarded as copies generated by polyploidization, or segmental duplication, while two or more AQP genes with the same chromosomal localization were defined as tandem duplication [56].

For the collinearity analysis, AQP genes from *T. urartu* were identified by alignment with BLASTn hosted in the EnsemblPlant database using the AQP gene sequences of the sub-genome A of wheat. Next, the location of the orthologue genes on the chromosome of *T. urartu* was obtained from the databases published by [57]. The syntenic relationships between AQP gene paralogs on each wheat chromosome, together with the collinearity map between wheat and *T. urartu*, were obtained using the web-based service ClicO FS [58].

### 2.4. Nonsynonymous Substitution Rate (Ka) and Synonymous Substitution Rate (Ks) Calculations

To determine the selection pressure that is exerted on duplicate AQP genes, we calculated the ratio Ka/Ks (purifying selection Ka/Ks < 1, neutral selection Ka/Ks = 1 and positive selection Ka/Ks > 1) [59]. To obtain the non-synonymous substitution rate and synonymous substitution rate of the AQP genes, the codon evolution rate was calculated using the duplicate genes previously found with the ClustalW codons by MEGA7 program [47] and using the neighbor-joining model [56]. The approximate date of the duplication events was estimated using T = Ks/2λ × 10^−6^ million years ago (Mya), based on the clock-like rates (λ) in grasses of 6.5 × 10^−9^ [60].

### 2.5. Expression Patterns of Aquaporin Genes in Several Organs and Developmental Stages and in Wheat Seedlings Growing Under Osmotic Stress

Microarrays data for wheat were obtained from IWGSC using the WheatEXP tool (https://wheat.pw.usda.gov/WheatExp/). The expression data for AQP genes were obtained in three developmental stages and five different organs (root, leaf, stem, spike and grain) according to the Zadoks scale [61,62]. For the analysis of expression patterns during osmotic stress, we used expression data of leaves of one-week-old seedlings grown in 20% PEG-6000 [15]. The magnitude of change in stress condition was expressed as the ratio compared to the initial condition (time zero). Expression profiles were grouped and presented as heatmaps [63].

### 2.6. Expression Analysis of Aquaporin Genes in a Drought-Tolerant and Susceptible Genotypes of Wheat Grown with and Without Water Stress during Grain Filling

Two genotypes of spring wheat (*T. aestivum* L.) with contrasting tolerances to water stress were selected according to the yield tolerance index (YTI) from a previous study [2]. One was advanced line with high yield potential under water stress conditions, “Fontagro 8” with a YTI of 0.60; and the other line, “QUP2569” with a lower YTI of 0.17, was considered as susceptible. The plants grew in a greenhouse, with a temperature range between 18 and 25 °C. 

Five seeds of each genotype were sown in 7.5 L pots of 26 cm in diameter and 21.1 cm height, filled with a 1:1:1 mixture of organic soil mix (Anasac, Talca, Chile), perlite and river sand, representing a total weight per pot of 4.9 kg. After the emergence of the second leaf, the seedlings were thinned to one per pot. Plants were fertilized until the tillering stage using Hoagland nutrient solution (PhytoTechnology Laboratories, Lenexa, KS, USA). All pots were irrigated at field capacity (CC) to anthesis stage (Z61) [61]. From anthesis onward, two water regimes were established, 100% CC (control) and without irrigation (water stress). The stomatal conductance (*gs*) was evaluated on three flag leaves of each replica between 11:00 and 15:00 h using an infrared gas analyzer (IRGA) CIRAS2 (PPSystem) (Table S1). The first sampling of leaves and roots were carried out when the *gs* of the treatment plants without irrigation reached about 50% of the control plants in each genotype, sampling 10 plants from the control treatment and 10 plants from the water stress treatment. Then, 20 pots were kept without irrigation and a second sampling was carried out when the *gs* of the stressed plants reached about 30% of the control plants (two days after the first sampling). The ten remaining water stress treatment pots were watered to CC and after two days a third sampling was taken. There were 60 replicates pots of each genotype (n = 120), considering two treatments and three sampling times. The samples collected were immediately frozen in liquid nitrogen and stored at −80 °C until analysis. 

RNA extraction from leaves and roots was done using the TRIzol^®^ reagent (Life Technologies, Carlsbad, CA, USA) and the manufacturer’s protocol. RNA concentration and quality were determined spectrophotometrically using the Infinite^®^ 200 PRO NanoQuant (Tecan, Männedorf, Switzerland). The integrity of the RNA was verified by agarose gel electrophoresis. Subsequently, the samples were treated with DNase Turbo™ Ambion^®^ (Life Technologies) according to the manufacturer’s instructions. The RNA concentration was then standardized to 25 µg µL^−1^ and finally the complementary DNA synthesis was performed using the First Strand cDNA Synthesis Kit (Thermo Scientific, Waltham, MA, USA) according to the protocol described by the manufacturer [64].

The gene expression of a selected suite of AQP genes was measured at the transcript level, by quantitative PCR (qPCR) using a Stratagene Mx3000p thermal cycler (Agilent Technologies, Santa Clara, CA, USA). The Maxima SYBR Green/ROX qPCR Master MIX kit (Thermo Scientific) was used for all reactions according to the protocol described by the manufacturer. For each sample (three biological replicates), qPCR was done in triplicate (three technical replicates), using 10 μL of Master MIX, 0.5 μL of 250 nM primers, 1 μL of cDNA and nuclease free water to a final volume of 20 µL. Amplification was followed by a melting curve analysis from 55 to 95 °C with continuous fluorescence measurement. Expression quantification was normalized using the *Ta-α-Tubulin* gene which is constitutively expressed in the organs analyzed [65,66]. The primers used for qPCR are described in Table S2. Transcript levels of each gene were evaluated using the 2^−ΔΔCT^ method [67]. The qPCR data was analyzed using ANOVA (one-way analysis of variance) tests (with a significance of α = 0.05). Data management and standardization were performed with R version 3.2.5 [68].

## 3. Results

### 3.1. The Wheat Genome has 113 Non-Redundant Aquaporin Genes

**Figure 1:**
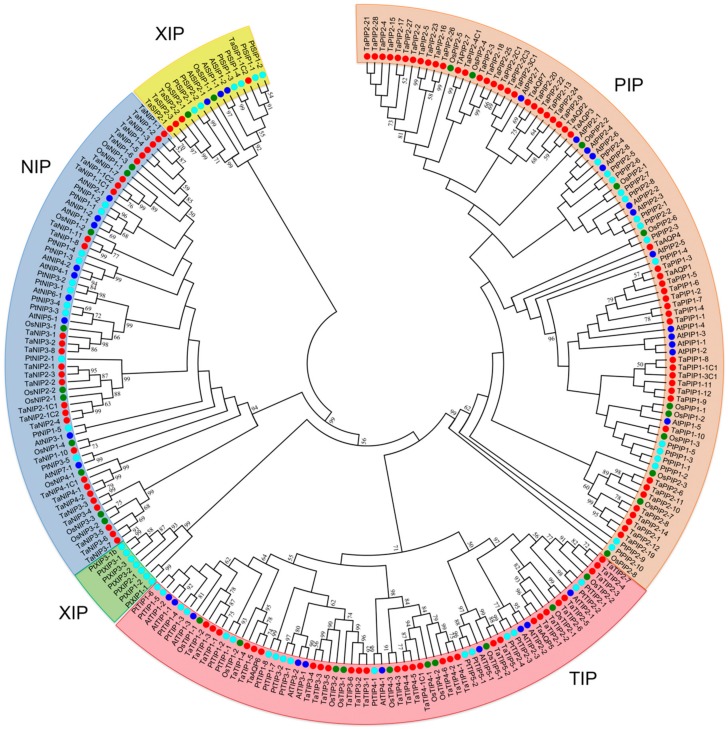
Phylogenetic tree for Aquaporins (AQP) identified in *Triticum aestivum*, *Arabidopsis thaliana*, *Populus trichocarpa* and *Oryza sativa*. Five protein families (PIP, NIP, TIP, XIP and NIP) were identified. In red circles, 113 AQPs from wheat; in light-blue, 56 from *P. trichocarpa*; in dark-blue, 36 from *A. thaliana*; in green, 33 from *O. sativa* (Table S4).

We obtained 123 sequences of non-redundant AQP gene homologs in the wheat genome. Among these, 10 pseudogenes were omitted from the subsequent analyses. Consequently, we used 113 AQPs (Table S3) to construct a phylogenetic tree together with the orthologue AQP sequences of *A. thaliana*, *O. sativa* and *P. trichocarpa*. The tree displayed five large families; all wheat sequences were grouped into the TIP, PIP, NIP and SIP families and no wheat AQP grouped in the clade XIP, composed only of *P. trichocarpa* AQPs. Altogether, 29 proteins were grouped in the TIP family, 52 in the PIP family, 4 in the SIP family and 28 in the NIP family. Similar proportions of members were established in related species, such as *H. vulgare* (Table S4). Then, we assigned each wheat AQP a name according to previously identified AQPs and their orthologues of *O. sativa* and *A. thaliana* (Figure 1, Table S5).

### 3.2. Each Aquaporin Subfamily Presents Particular Physicochemical and Structural Characteristics

For the 113 wheat AQPs proteins, basic physicochemical characteristics were analyzed such as molecular weight, isoelectric point, subcellular localization, conserved motifs and amino acids associated with the selectivity pore, among others (Table S3). Generally, the AQP proteins had molecular weights (MWs) ranging from 19.3 to 47.2 kDa and isoelectric points (IPs) between 5.33 and 9.82. At the sequence level, 82 AQP proteins had 6 transmembrane domains and the remaining had 4, 5 or 7 domains (Table S6). Subcellular localization prediction for the AQP proteins indicated that they are located in the plasma membrane (59.3%), tonoplast (22.1%), chloroplast (11.5%), mitochondria (3.5%), endoplasmic reticulum (2.7%) and peroxisome (0.89%). Regarding the conserved NPA motifs, 106 AQP proteins maintained the sequence of the first conserved motif and 110 the second NPA motif. The ar/R selectivity filter present in the PIP family was conserved in all its members, while in the TIP family the filter was conserved at the subfamily level. On the other hand, the NIP family presented the greatest variations. For example, TaNIP1-11 and TaNIP1-10 change their amino acids in the first and second positions, respectively, while in the NIP3 subfamily only the amino acid at the fourth position was conserved among all their members (Table S3).

### 3.3. Gene Structure and Conserved Motifs of Wheat Aquaporin Proteins Confirm the Phylogenetic Classification

**Figure 2:**
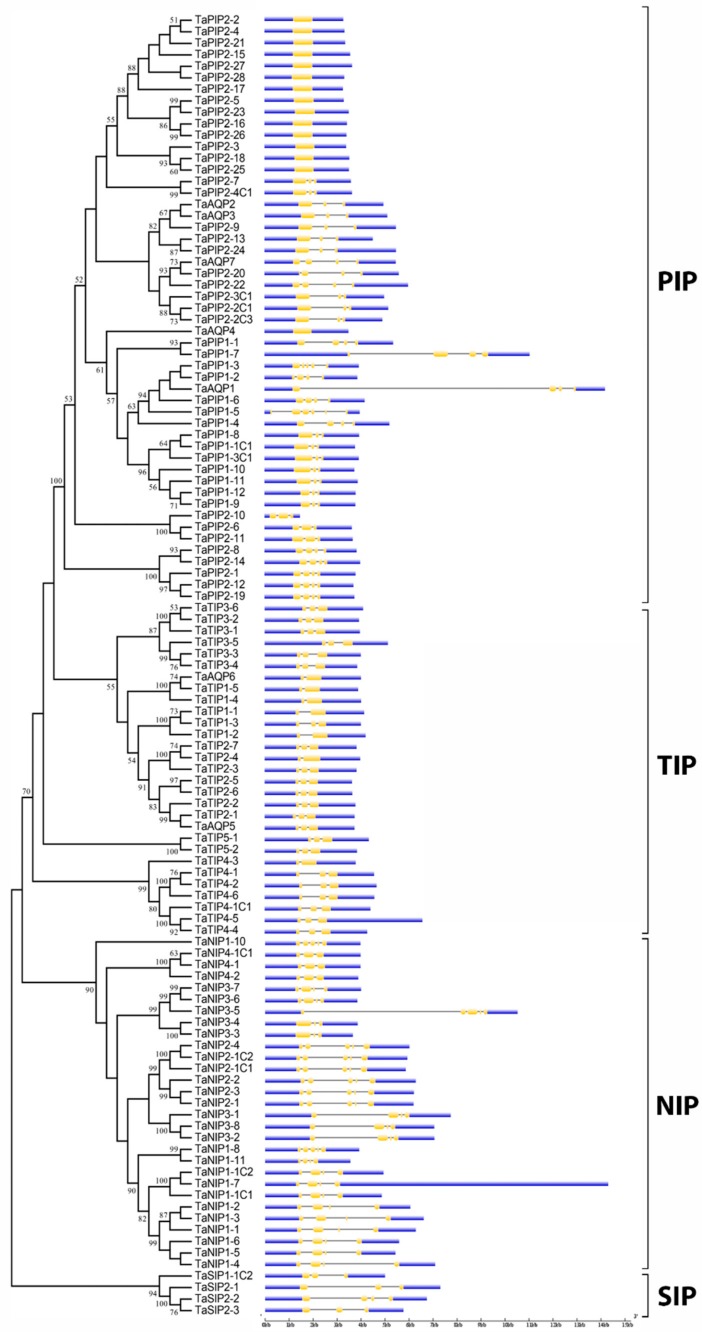
Exon-intron structure of wheat AQPs. In yellow, exons; in blue, the 3′ and 5′ UTR regions; and in the grey lines, introns.

**Figure 3:**
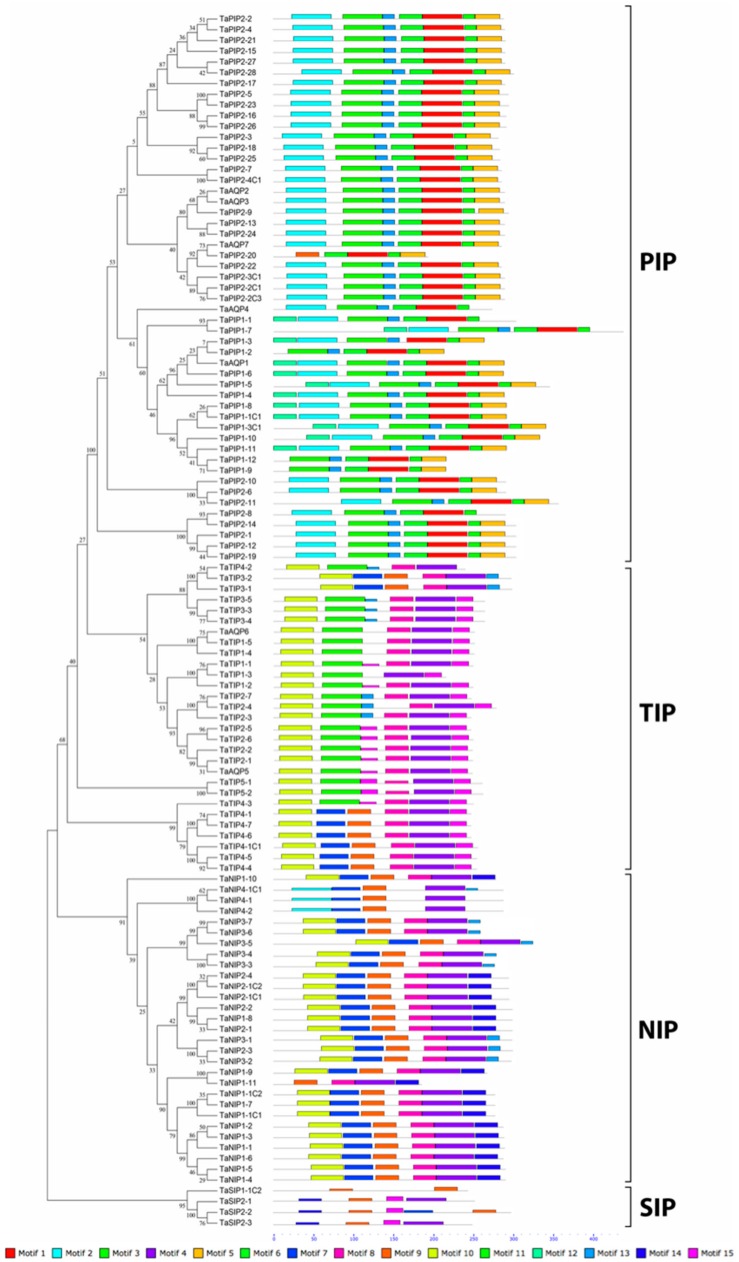
Identification of conserved motifs in wheat AQPs. The MEME database identified 15 motifs using the complete amino acid sequence represented proportionally by grey lines. Each color represents a different motif described in Table S7.

Gene exon-intron structural and motif analyses are relevant for the evolutionary study of gene families and to support the phylogenetic tree. The distribution of introns and exons in the 113 AQP genes was analyzed, revealing that the divergences in gene structures are consistent with phylogenetic classification (Figure 2, Table S7). All TIP genes had 1 to 2 introns. Within the PIP family, the PIP1 subfamily had 2 to 5 introns and the longest intron of all AQPs found, *TaAQP1* 10.5 kb. A significant number of PIP2 subfamily members had not introns and only some cases had 2 to 3 introns. The NIP family had 2 to 4 introns and the SIP family had 2 to 3. In general, the length and number of introns within each family or subfamily of AQP was similar to those of related species, such as *H. vulgare*. Then, to strengthen our phylogenetic analysis, a strategy for MEME analysis was also developed. Fifteen motifs were identified, named from 1 to 15 (Figure 3). Among these, the sequences of motifs from 1 to 10 matched with AQPs domains according to the analysis with InterPro Scan 5 (Table S8). The different motifs were grouped correspondingly with each big family or subfamily. Thus, similar domain patterns supported the relationships and phylogenetic classification of wheat AQP proteins.

### 3.4. Segmental Duplications of Aquaporin Genes Have Contributed to High Number of Members in the Plasma Membrane Intrinsic Protein Family

**Figure 4:**
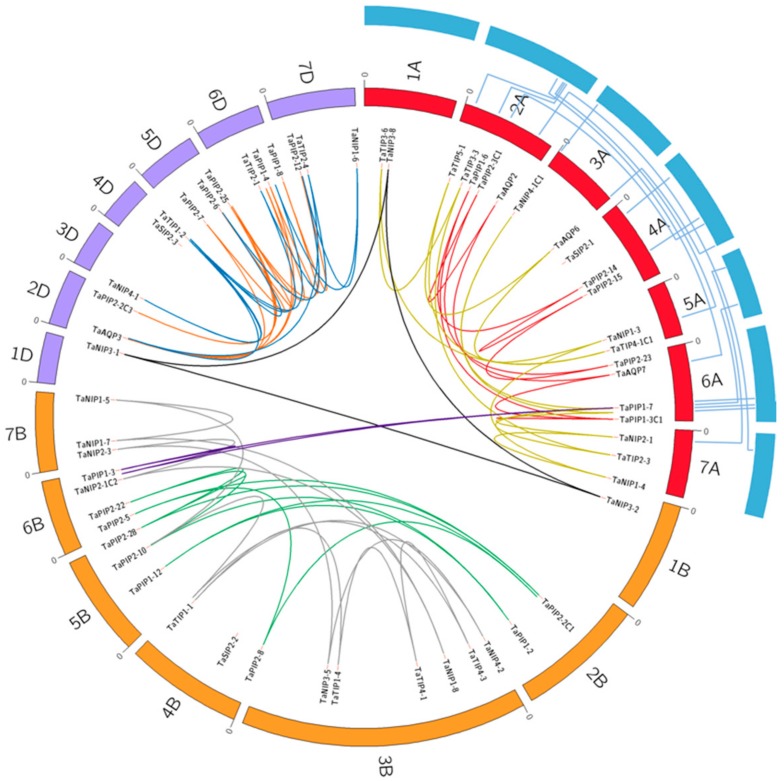
Analysis of synteny and chromosomal distribution of duplicated AQP genes of *T. aestivum* homolog groups and collinearity among AQP genes from *T. aestivum* and *T. urartu*. The locations of AQP genes are indicated on respective A, B and D sub-genomes of *T. aestivum*. Eight paralog groups are displayed in different colors. The light blue on the periphery represents chromosomes of *T. urartu*. The collinearity among TuAQPs and TaAQPs genes is represented by the lines between orthologue gene-pairs.

From the 113 AQP genes identified, the chromosomal location of 76 genes was determined and 8 clades of paralog genes were established (Figure 4, Table S9). Among them, 35 genes were located in sub-genome A, 42 in B and 36 in D. All chromosomes include AQP genes. The sets of chromosomes 7 and 1 were those with higher and lower abundance of genes, with 21 and 6 AQP genes, respectively. Polyploidization events play an important role in the expansion of the AQP genes in wheat, whose origins go back to the hybridization events between the species of *T. urartu*, *A. speltoides* and *A. tauschii* [69]. In this way, wheat had 24 groups of genes that were homolog copies on each of the A, B and D chromosomes, 15 pairs were on the A, B or D chromosomes and 11 genes were unique copies. Among them, *TaAQP3* – *TaPIP2-24* and *TaPIP2-27* – *TaPIP2-28* were tandem duplications and 21 pairs were segmental duplications concentrated mainly in the PIP family (Table 1 and Table S10). In the same context, to determine if the array of genes of the AQP family on the D sub-genome of wheat was conserved with respect to the genome of *T. urartu*, 24 pairs of orthologue genes were identified. Interestingly, some of these genes could be associated with the abiotic stress tolerance capacity of *T. urartu*. Among them, 8 genes maintained their chromosomal location in respect to wheat genome (Figure 4, Table S11), evidencing a medium collinearity level between the AQP genes of both species. This suggests that about half of the genes have changed their chromosomal location in the D sub-genome due to chromosomal inversion and crossover events.

To determine possible evolutionary forces acting on wheat AQP genes, the Ka/Ks ratio (synonymous and nonsynonymous substitutions per site) was estimated for each pair of duplicated genes (Table 1). Practically all the Ka/Ks ratios were less than 1 (between 0.05 and 0.84), suggesting that the purifying selection has an important role in maintaining the function of the AQP genes in wheat. Only on the pair *TaPIP2-18* – *TaPIP2-3* (Ka/Ks = 1.38) a positive selection pressure has been exerted. We also calculated the time of divergence between these duplicated genes and found that the duplications of the AQP genes were between 6,08 and 40.31 million years ago (mya), indicating that these duplications in the AQP family in wheat occurred before of the divergence of the common ancestor of the Triticum-Aegilops group (~4–5 mya) [70].

### 3.5. Spatio-Temporal Expression of Aquaporin Genes in Wheat

**Figure 5:**
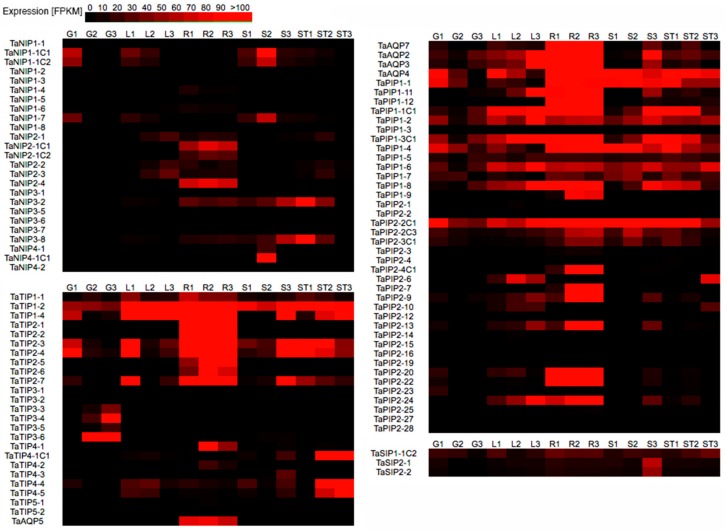
Analysis of AQP expression in different organs and developmental stages of wheat. Each heatmap corresponds to a subfamily of AQP. The intensity of the red color indicates the level of up-regulation of the gene in FPKM values. Development stages are indicated according to Zadoks scale: G1: Grain_Z71; G2: Grain_Z75; G3: Grain_Z85; L1: Leaf_Z10; L2: Leaf_Z23; L3: Leaf_Z71; R1: Root_Z10; R2: Root_Z13; R3: Root_Z39; S1: Spike_Z32; S2: Spike_Z39; S3: Spike_Z65; ST1: Stem_Z30; ST2: Stem_Z32; ST3: Stem_Z65. Microarray data were obtained from [62].

Analysis of gene expression profiles is a useful tool to understand their biological functions. Therefore, we analyzed the expression patterns of wheat AQP genes using microarray data obtained from public databases. In this approach, 94 of the 113 AQP genes (83.18%) were identified and a heatmap was designed to evaluate the abundance of transcripts in roots, leaves, stem, spikes and grains according to the Zadoks growth scale (Figure 5, Table S12). We observed that all AQP genes were expressed in at least one organ, with the exception of *TaTIP5-2*, indicating that they had remarkable organ and developmental specificity. For example, some NIP genes had high transcript levels in spikes, roots and stems, as *TaNIP1-1C1*, *TaNIP1-1C2*, *TaNIP1-7*, *TaNIP4-1C1*, or *TaNIP2-1C1*, among others. In the case of the TIP family, some TIP1 and TIP2 subfamily genes were expressed in the three stages of root, leaf, spike or stem development. Transcripts of the PIP family were accumulated in all the analyzed organs and developmental stages. Most of the members of this family were expressed in all developmental stages of roots and in one or two stages in leaf, stem, spike or grain. Within this family, *TaAQP7*, *TaTIP1-12* and *TaTIP2-22* were specifically expressed in roots, whereas *TaPIP1-5* had constitutive levels in all organs and stages of development. On the other hand, *TaPIP1-6* accumulated at different degrees in all organs and stages evaluated. In the SIP family only *TaSIP1-1C2* transcripts accumulated in all organs, unlike *TaSIP2-1* and *TaSIP2-2*, which were observed only in spikes.

### 3.6. Wheat Aquaporin Gene Expression in Response to Osmotic Stress

**Figure 6:**
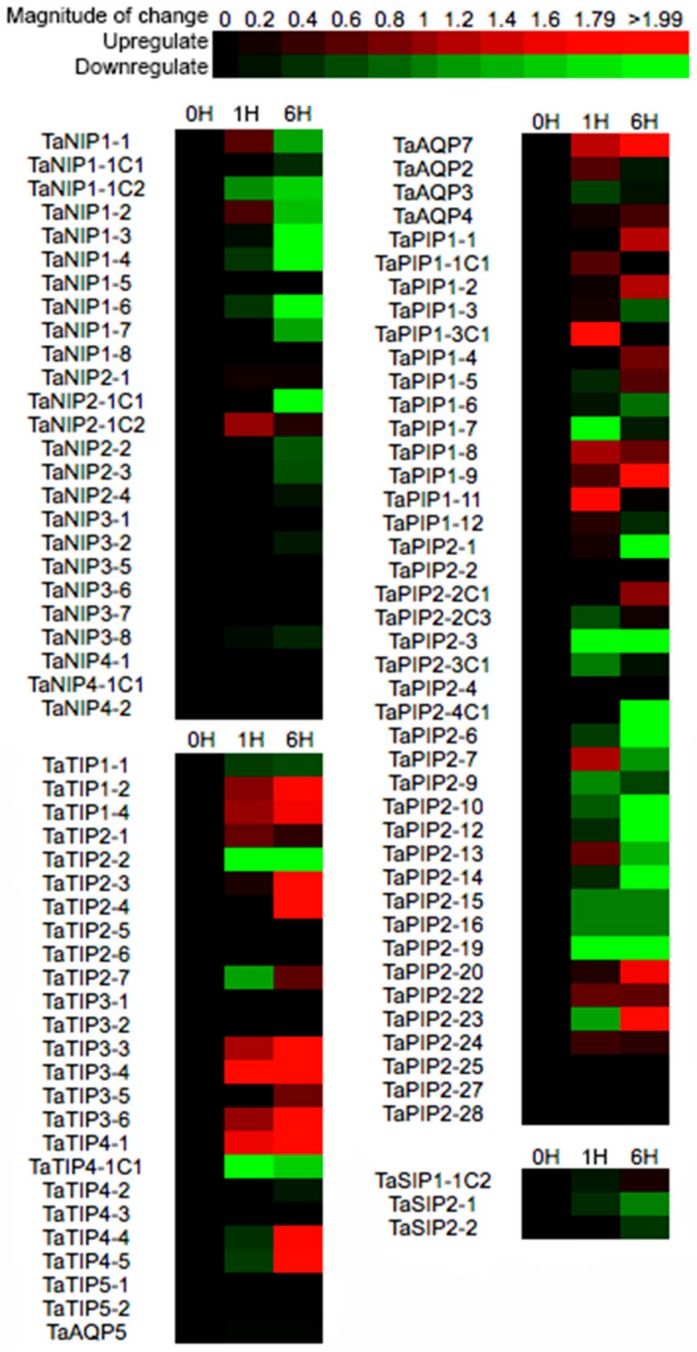
Analysis of AQP gene expression of wheat seedling leaves under osmotic stress. Green indicates a negative change or down-regulation and red indicates a positive change or up-regulation at 1 or 6 hours after treatment. The intensity of the colors indicates the fold change. Microarray data were obtained from [14].

In order to understand the role of AQP genes in stress under water deficit in wheat, we analyzed their expression patterns under osmotic stress, using microarray data obtained from WheatExp database (Figure 6, Table S13). The results showed that osmotic stress regulated differentially the accumulation of AQP transcripts. NIP genes tended to be down-regulated after 6 hours of stress. In the NIP1 subfamily, *TaNIP1-3*, *TaNIP1-4* and *TaNIP1-6* were strongly repressed, as was *TaNIP2-1C1* from the NIP2 subfamily. Similarly, the expression of SIP genes was also down-regulated. In contrast, the transcripts of the majority of the TIP family increased (12 of 25 genes) at 6 hours of stress, with the exception of *TaTIP1-1*, *TaTIP2-2* and *TaTIP4-1C1* that were repressed. On the other hand, in the PIP family, 17 genes were up-regulated and 16 down-regulated at 6 hours of stress, whereas the expression levels of 13 genes were not altered. In this case, most down-regulated genes belonged to the PIP2 family (13 genes). In summary, we observed that some genes highly expressed in leaves or roots, such as: *TaPIP1-1*, *TaPIP1-3C1*, *TaAQP7*, *TaPIP2-2C1*, *TaTIP2-24*, *TaPIP1-2*, *TaTIP2-3*, *TaTIP2-4*, or *TaTIP4-1*, were also highly induced during stress, suggesting that they could be associated with the drought tolerance capacity of wheat.

### 3.7. Expression Analysis of Aquaporin Genes in Drought-Tolerant and Susceptible Wheat Genotypes Grown with and without Water Stress during Grain Filling

**Figure 7:**
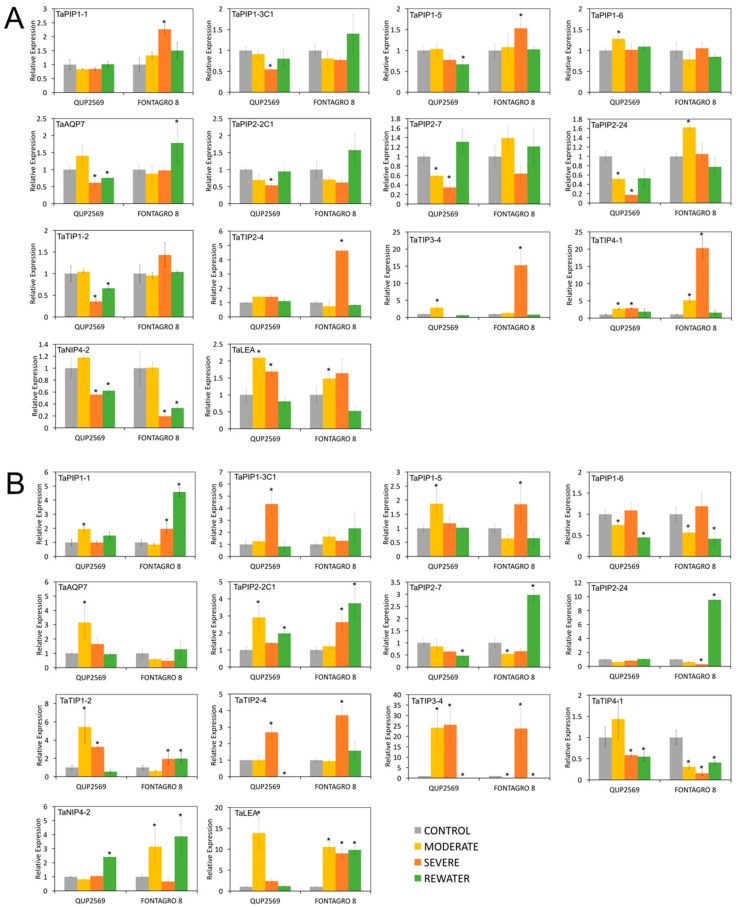
Expression profiles of AQP genes for drought-tolerant (Fontagro 8) and drought-susceptible (QUP2569) genotypes with and without water stress. Quantitative PCR analyses for 13 AQP genes in leaves (**A**) and roots (**B**) of wheat genotypes under well-watered conditions (control), moderate water stress, severe water stress and re-watering. Columns and error bars represent the mean and standard deviation for three biological by three technical replicates. * *p*-value < 0.05.

To investigate in more depth the relationship between the expression patterns of AQP genes and the tolerance capacity of wheat, we performed an analysis of relative expression of some genes previously mentioned by qPCR in leaves and roots between two wheat genotypes (accessions) with contrasting tolerance responses to water deficit (Figure 7). In this sense, we observed important differences at the transcript level between the drought-tolerant (Fontagro 8) and the drought-susceptible genotype (QUP2569). In leaves, as stress intensity increased, some AQP genes of the PIP1 and PIP2 subfamilies, like *TaPIP1-1*, *TaPIP1-5*, or *TaPIP2-24*, were clearly up-regulated in the tolerant genotype, whereas in the genotype susceptible the expression levels were down-regulated (Figure 7A). In the case of the TIP family, they also increased their expression levels during severe stress. On the other hand, an opposite effect was seen in the expression of AQP genes in roots, where the tolerant genotype showed a down-regulation of *TaPIP1-1*, *TaPIP1-5*, *TaAQP7*, *TaPIP2-1C1*, *TaTIP2-1*, *TaTIP3-4* and *TaTIP4-1* genes under moderate stress, while the susceptible one presented up-regulation (Figure 7B). Interestingly, during the rehydration of the tolerant genotype, several genes of the PIP2 subfamily were induced in leaves and roots. This could be associated with a better internal water balance and faster rehydration ability of Fontagro 8.

## 4. Discussion

The increasing number of sequenced genomes opens new horizons in the study of functional genomics and the precision of annotating new genes [71,72]. In this sense, genome-wide identification methods allow greater accuracy in the characterization of multiple gene families, such as AQPs. These play an important role in the cellular homeostasis of different organs and tissues, increasing the permeability of the plasma membrane to water and essential nutrients [2,33]. Different studies in species like *O. sativa* [73], *Z. mays* [74], *Sorghum bicolor* [75], *Setaria italica* [76], or *H. vulgare* identified AQPs in cereals. In addition, previously, 48 AQP genes [35,36] were identified in wheat by EST analysis. In this study, 65 new non-redundant AQP genes were identified, a number that is consistent with the allohexaploid genome, as predicted earlier by Forrest and Bhave [77], who suggested that a higher number of AQP genes are present in the wheat genome.

Consistent with the AQP classification of *O. sativa* [73], *H. vulgare* [23], *A. thaliana* [78] and *P. trichocarpa* [79], the 113 wheat AQP genes total grouped in 4 families: NIP, TIP, PIP and SIP. At the same time, NIP separated according to the pattern of conserved motifs of 5 subfamilies: NIP1, NIP2, NIP3, NIP4 and NIP5; PIP of 2 subfamilies: PIP1 and PIP2; TIP of 5 subfamilies: TIP1, TIP2, TIP3, TIP4 and TIP5; and SIP of 2 subfamilies: SIP1 and SIP2. The structure of these groups is closely related to the genetic relationships with *O. sativa*, *A. thaliana* and in a lower degree with *P. trichocarpa*, which supports previous phylogenetic classifications [22,80,81]. Coincidentally, the structural exon-intron patterns of the genes of all subfamilies of wheat AQPs are specific to each subfamily and similar in number and length only to closely related species, such as *H. vulgare* [23]. It would reflect a high degree of conservation for this gene family, despite the processes of natural and artificial polyploidy that occurred in wheat [82].

By evolutionary analyses, polyploidy events and segmental and tandem duplications have had an important role in increasing the total number of genes [82,83]. In wheat, each sub-genome provides approximately one-third of the family of AQPs. However, 20% are genes duplicated in tandem or segmentally, indicating that both mechanisms have contributed significantly to the expansion of the members of this family, especially of the PIP family. A similar result has been reported in *B. rapa* [71] and *Z. mays* [74]. During evolutionary processes, events of crossover and inversion of important chromosome zones, even distal, can modify the location and function of hundreds of genes [84]. In this context, the collinearity levels between the orthologue AQP genes of the wheat D sub-genome and *T. urartu* reveal that their localization has been conserved, which is consistent with the *auxin/indole-3-acetic acid* family of genes in wheat [56]. However, some pairs of orthologue genes (e.g., *TaAQP6* or *TaAQP7*) present different locations between both genomes, suggesting re-arrangements throughout the evolution of the AQP gene family in wheat, similar to that evidenced by the *MADS*-*box* family of this same species [85]. The different duplications identified in this work reached values of Ka/Ks ratio <1, indicating that the evolutionary history of the AQP genes is under a purifying selection pressure, which suggests a high conservation of the duplicated genes, similar to the AQP family in *B. rapa* [71]. Taken together and despite the structural redundancy, it is evident that for several pairs of duplicated genes (for example, *TaPIP1-12* and *TaPIP1-1C1*) the patterns of organ expression and specific time of expression are different, eventually revealing divergence and functional diversity [86]. This is interesting because it suggests that functional changes are often regulated by genetic and epigenetic interactions between homoalleles, providing a sufficient amount of plasticity required for coping with new environmental scenarios [87].

The microarray data analysis revealed that a large number of AQP genes, unique or duplicates, especially PIP and TIP, are highly expressed in wheat leaves, consistently with homolog genes reports in other species [29,88,89,90] and confirmed here with the expression analysis. The comparison of two contrasting wheat genotypes growing in greenhouse conditions with and without water stress during grain filling revealed different AQP expression patterns in leaves and roots between the tolerant and the susceptible genotypes. In the tolerant genotype, the relative abundance of transcripts for different AQP genes in roots decreased under moderate water stress, while that in the susceptible one it increased. Interestingly, this inverse pattern is similar to that reported in contrasting *O. sativa* genotypes, a monocotyledonous species related to wheat [91]. On the other hand, we observed that in the tolerant genotype, the expression levels of several PIP1, PIP2 and TIP genes increase during the re-watering. It is probably that the flow of water mediated by PIP1 or PIP2 towards the cytoplasm and by TIPs towards the vacuole in tolerant genotype is efficiently conducted for its compartmentalization. This hypothesis agrees with studies of PIP1 from *Salicornia bigelovii* or *AQP7* from wheat expressed in *Nicotiana tabacum*, *PgTIP1* from *Panax ginseng* in *Glycine max* and *SlPIP2-7* from *S. lycopersicum* or *JcPIP2-7* from *Jatropha curcas* in *A. thaliana* [25,32,92,93,94]. However, there is no total consensus about the physiological mechanisms associated with the function of AQP genes and how and/or why these genes are up or down-regulated in plants under the same water stress [95]. Different studies have associated the down-regulation of AQPs with an adaptive mechanism to reduce the effects of water deficit stress [96,97] but future analyses associated with the functional characterization of these candidate genes or proteins will be necessary to confirm these results and to draw general conclusions.

## 5. Conclusions

The breeding of wheat cultivars with augmented tolerance to water deficit requires, among other subjects, an exhaustive characterization of the molecular mechanisms that underlie physiological processes in the plant. The characterization of proteins related to the control of water status in tissues and organs, from a structural and functional genomic point of view, could contribute to this goal. The results obtained in this study updated the classification of AQP genes and proteins recently generated by several sequencing projects. Furthermore, they showed that, in spite of the presence of a high number of AQP genes in wheat (113), an important number of them are copies coming from polyploidization events and from segmental or tandem duplications in the ancestral species (60%). Although the conservation rate is also high, there is functional diversity as revealed by the expression patterns of several copies of AQP genes in different organs and between contrasting genotypes when they are subjected to stress by water deficit. Thus, this research contributes relevant information for future studies on wheat AQPs and confirms the complexity associated with their regulation. 

## Figures and Tables

**Table 1:** Evolutionary parameters for duplicated AQP genes. Table shows Ka/Ks ratios, duplication and selection types and time of divergence.

AQP Pairs			Duplication Type	Ka ^1^	Ks ^2^	Ka/Ks	Selection	Time (mya ^3^)
*TaAQP1*	vs.	*TaPIP1-2*	Segmental	0.03	0.08	0.34	purifying	6.08
*TaAQP2*	vs.	*TaPIP2-13*	Segmental	0.02	0.16	0.09	purifying	12.54
*TaPIP2-9*	vs.	*TaPIP2-24*	Segmental	0.02	0.14	0.13	purifying	10.85
*TaNIP1-3*	vs.	*TaNIP1-4*	Segmental	0.06	0.18	0.32	purifying	13.77
*TaNIP1-2*	vs.	*TaNIP1-5*	Segmental	0.04	0.15	0.25	purifying	11.54
*TaNIP1-1*	vs.	*TaNIP1-6*	Segmental	0.06	0.20	0.28	purifying	15.23
*TaPIP1-11*	vs.	*TaPIP1-3C1*	Segmental	0.01	0.12	0.05	purifying	9.15
*TaPIP1-10*	vs.	*TaPIP1-12*	Segmental	0.01	0.27	0.05	purifying	20.38
*TaPIP2-1*	vs.	*TaPIP2-14*	Segmental	0.05	0.13	0.41	purifying	10.23
*TaPIP1-12*	vs.	*TaPIP1-1C1*	Segmental	0.01	0.20	0.06	purifying	15.46
*TaPIP1-9*	vs.	*TaPIP1-8*	Segmental	0.01	0.22	0.06	purifying	16.62
*TaPIP2-11*	vs.	*TaPIP2-1*	Segmental	0.23	0.36	0.63	purifying	27.69
*TaPIP2-10*	vs.	*TaPIP2-19*	Segmental	0.27	0.33	0.82	purifying	25.15
*TaPIP2-6*	vs.	*TaPIP2-12*	Segmental	0.27	0.32	0.84	purifying	24.46
*TaPIP2-19*	vs.	*TaPIP2-8*	Segmental	0.16	0.22	0.71	purifying	17.00
*TaPIP2-17*	vs.	*TaPIP2-28*	Segmental	0.03	0.22	0.12	purifying	16.69
*TaPIP2-4*	vs.	*TaPIP2-2*	Segmental	0.04	0.24	0.15	purifying	18.38
*TaPIP2-28*	vs.	*TaPIP2-27*	Tandem	0.02	0.27	0.06	purifying	20.38
*TaPIP2-27*	vs.	*TaPIP2-21*	Segmental	0.02	0.18	0.09	purifying	13.77
*TaPIP2-26*	vs.	*TaPIP2-23*	Segmental	0.18	0.29	0.60	purifying	22.46
*TaPIP2-16*	vs.	*TaPIP2-5*	Segmental	0.17	0.25	0.69	purifying	18.92
*TaPIP2-18*	vs.	*TaPIP2-3*	Segmental	0.72	0.52	1.38	positive	40.31
*TaAQP3*	vs.	*TaPIP2-24*	Tandem	0.02	0.14	0.11	purifying	10.85

^1^ Ka: number of nonsynonymous substitutions per non-synonymous site; ^2^ Ks: number of synonymous substitutions per synonymous site. ^3^ Mya: million years ago.

## Supplementary Materials

The following are available online at http://www.mdpi.com/2073-4425/9/10/497/s1, Table S1: Evaluation of physiological parameters of wheat contrasting genotypes under water stress, Table S2: Primers used in this study, Table S3: Characterization of genes identified in this study, Table S4: Number of proteins in each family and subfamily of aquaporins in different species, Table S5: Accession codes of AQP genes used in the phylogenetic tree, Table S6: Predicted transmembrane domains of AQP proteins, Table S7: Pattern of introns and exons in *T. aestivum* and *H. vulgare*, Table S8: Regular expression profile of the conserved motifs defined by MEME of AQPs, Table S9: Chromosomal location and paralog groups of AQP genes, Table S10: Homologs and duplications of AQP genes, Table S11: Pairs of orthologue genes identified in *T. aestivum* and *T. Urartu*, Table S12: Analysis of AQP gene expression in different organs and stages of wheat development, Table S13: Analysis of AQP gene expression in wheat seedlings responding to osmotic stress.
